# Diphtheria

**Published:** 1884-11

**Authors:** 


### DIPTHERIA.

THE disease known as Diptheria, which is clearly analagous to the old-fashioned putrid sore throat, is extending its ravages from month to month and from year to year, and may fairly be looked upon as among the most potent causes of mortality at present known to this and other communities. Consumption, dysentery and pneumonia still take the lead, in the order named, but diptheria follows closely upon these, and is likely to eclipse them all: since science is ever gaining added control over the three most deadly ailments known to our latitude, while diptheria steadily increases in malignity, and in the power to destroy. The present rate of mortality from diptheria is hardly less than 3,000 a year in this city alone. In London, with three times the population, the yearly mortality from this disease has never exceeded five hundred. It becomes us to ask why New York should have more deaths from this fell-destroyer, according to population, than the great British metropolis?

If we were comparing London and Peking we might give a quick solution to the problem in the different conditions of medical and sanitary science. We should only consider it necessary to mention the fact that medical literature is scarcely known in China, and that the poor barbarians of the flowery kingdom can hardly be expected to do much in the way of saving human life from destruction.
But this plea cannot be interposed in behalf of the American metropolis. We have as able and as learned physicians in New York as can be found in Europe, and there are no methods of treatment known to the profession in any part of the world which are unknown and untested among us. Clearly, then, the immunity of the British metropolis is not due to superior medication.

To what, then, shall it be charged? We answer, to a variety of causes, which, combined, are ample to produce the result. Prominent among these is our defective system of drainage and sewerage. The greatest mortality has been in localities which were once low and marshy—in short, in regions of "madeland." The filling used has served to cover from view the soft, moist soil, but it has not neutralized the noxious vapors which escape from it. In these localities are situated the tenement dwellings of the city, for the occupants of which, poor food, bad air and imperfect clothing ably supplement the deleterious exhalations. In these localities the disease after assumes the proportions of an epidemic, and its contagious character is clearly established. If, under these circumstances, so favorable to its extension, a case occurs, the proportion of those who suffer from exposure to the contagion is nearly or quite as great as that of those who succumb after exposure to the small-pox, while the rate of mortality is unquestionably greater. The difference between the perfectly-drained and admirably sewered London streets and our own sham system is sufficient to account for a reasonable proportion of the excessive mortality to which we have alluded.

But there are other localities which suffer in even greater ratio than the city of New York. There is hardly a settlement, however small, in the Eastern and Middle States, which can be deemed free from this scourge. It is folly to attribute its growth to any single cause. Whatever tends to lessen human vitality, to lower the tone of the system, to deteriorate the vital fluids, may be said to encourage the disease. We are convinced that no cause exists more prolific in diptheritic results than carefully closed rooms, heated with stoves, furnaces and steamcoils. Of these, the last-named devices are the worst, because no provision whatever is made for a change of air. The furnace heat may and should be merely cool, pure, out-door air, heated by passing through a heating-chamber. This can do no harm, because it is not likely to be de-vitalized in the transit. But even under favorable circumstances like these, ventilation is important, and should never be neglected for a single hour out of the twenty-four.

Sunlight, too, is vastly important. We have seen those members of a family, whose chamber windows looked northward, taken down with diptheria, while the others, whose bed-rooms enjoyed a sunny outlook were wholly untouched.

Cleanliness erects very strong barriers against this disease. The man or woman, or boy or girl, who frequently washes himself or herself all over, using soap and warm water, and then rubs the surface till it is not only dry but in a glow—the whole process being completed in four or five minutes—will probably escape diptheria in any violent form, even though exposed to the contagion. So he who lives largely in the open air, exercising freely, will rarely take the disease, and will speedily convalesce when seized.

Good air, abundant exercise, cleanliness, suitable clothing, and wholesome food, are the best shields and safe-guards against this disease. It is, however, important to know how to arrest it quickly on its appearance.

The method which we have employed is simple and effective. It is merely the application of ice to arrest the local symptoms, and the use of fresh milk, abundantly, as food. It is surprising to observe the speedy results of this treatment. The swelling of the glands of the neck and under jaw, quickly disappears, and the profuse and offensive exudations from the mouth and throat and nostrils cease as by magic. The congestion and engorgement of the parts is relieved : the heat is diminished : the fetor is quickly lessened : the pulse is lowered, and the patient, sustained by new milk, recovers rapidly. We apply pounded ice in cloths to the swollen neck and jaws, and feed crumbs of it to the patient unceasingly. No attack which we have seen, has withstood this treatment for twelve hours. Other conditions, must of course be compelled : the feet must be placed in hot baths : the air must be pure and abundant, and sunshine must be availed of if practicable. But with proper attention the ice-treatment will almost invariably work a speedy cure. Lime-water Spray, discharged upon the throat and fauces, is relied upon by many medical men in diptheria, and it appears to ameliorate the diseased condition. But this is unneccessary where ice is employed in the manner which we have indicated.—The same has proved true in our experience with regard to solutions of iron, tannin, the acids, and all forms of astringents For the dangerous period, ice is our sole application.

